# Long-Range Correlation in Synchronization and Syncopation Tapping: A Linear Phase Correction Model

**DOI:** 10.1371/journal.pone.0007822

**Published:** 2009-11-13

**Authors:** Didier Delignières, Kjerstin Torre, Loïc Lemoine

**Affiliations:** Motor Efficiency and Deficiency Laboratory, University Montpellier I, Montpellier, France; Victoria University of Wellington, New Zealand

## Abstract

We propose in this paper a model for accounting for the increase in long-range correlations observed in asynchrony series in syncopation tapping, as compared with synchronization tapping. Our model is an extension of the linear phase correction model for synchronization tapping. We suppose that the timekeeper represents a fractal source in the system, and that a process of estimation of the half-period of the metronome, obeying a random-walk dynamics, combines with the linear phase correction process. Comparing experimental and simulated series, we show that our model allows accounting for the experimentally observed pattern of serial dependence. This model complete previous modeling solutions proposed for self-paced and synchronization tapping, for a unifying framework of event-based timing.

## Introduction

Finger tapping has been for a long time studied to elucidate the timing processes that underlie the production of rhythmic behavior. The most popular model for self-paced tapping was proposed by Wing and Kristofferson [1]. This model supposes that timing is controlled by an internal timekeeper that provides periodic events that trigger motor responses (taps). Each tap is performed after a motor delay, and the series of intervals produced by the timekeeper, as well as the successive motor delays are both considered as uncorrelated white noises. This simple model supports a set of easily testable hypotheses: (1) the timekeeper variance should present a Weberian increase with target interval length, (2) motor variance should be independent on target interval length, and (3) inter-tap interval series should present a negative lag-one autocorrelation, bounded to −0.5. These hypotheses were successfully tested in experiments during which participants produced series of 30–50 successive taps following different initially prescribed tempi [1], [2].

Vorberg and Wing [3] proposed to extend the initial model to account for tapping in synchronization with a periodic metronome. Their model supposes the existence of an auto-regressive correction process, correcting the current interval produced by the timekeeper by a fraction of the preceding asynchrony. This so-called *linear phase correction model* received empirical support from experiments where participants tapped in synchrony with a metronome for 30 to 50 successive taps.

Recently, a set of studies focusing on the analysis of longer series (i.e. hundreds of successive taps) provided new insights about the true nature of timing. Gilden, Thornton and Mallon [4] showed that series of intervals produced in self-paced tapping contained long-range correlations, close to 1/*f* noise (see also [5]). Gilden et al. [4] concluded that the central timekeeper should be considered a 1/*f* source, rather than white noise as postulated by Wing and Kristofferson [1]. Chen, Ding, and Kelso [6] showed that in synchronization tapping, the pattern of correlation in interval series was completely modified: in this task the series of intervals presented anti-persistent correlations, and in contrast persistent long-range correlations were found in the series of asynchronies to the metronome. Torre and Delignières [7] showed that incorporating a fractal source in the timekeeper component of the Vorberg and Wing [3]'s model allowed generating simulated series of asynchronies and inter-tap intervals that reproduced the experimentally observed dynamical signatures.

In another experiment, Chen et al. [8] analyzed serial dependence in synchronization (on the beat) and in syncopation (off the beat) tasks. They found persistent long-range dependence in asynchrony series in both conditions, but the strength of correlations was significantly higher in syncopation. Our aim in the present paper was to show that a simple extension of the original linear phase correction model [3] allows to account for the results obtained in syncopation, and especially for the increase in long-range correlation. We first describe in details the models proposed for accounting for self-paced and synchronization tapping, before presenting an extension for syncopation.

The model proposed by Wing and Kristofferson [1] supposes that the production of each interval is based on two independent processes: an internal clock, which provides a series of temporal intervals *C_i_*, and a motor component, responsible for the execution of the tap *i* at the expiration of the interval *C_i_*. This motor component does not operate instantaneously, and all taps have an assigned motor delay *M_i_*. The observed period *I_i_* then depends on both components
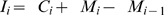
(1)


**Figure 1:**
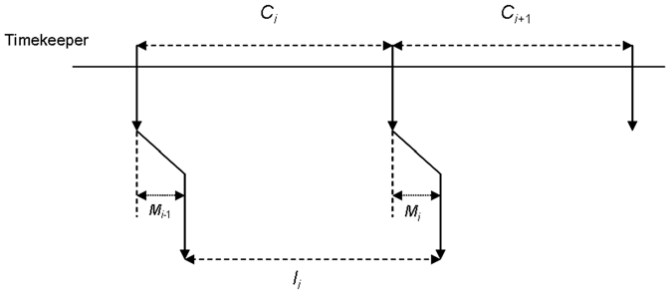
The Wing-Kristofferson model for self-paced tapping. *C_i_*: interval produced by the timekeeper, *M_i_*: motor delay, *Ii*: effective interval produced by tapping. We suppose in the present paper that *C_i_* presents fractal fluctuations.

The combination of the components of the model is illustrated in Figure 1.

The activation-threshold model [9] represents the simplest way for conceiving such an internal timekeeper. In this model an activation level is supposed to increase linearly in time. The attainment of a particular threshold determines a first event, and resets the activation level. The iteration of this simple process produces a succession of periodic events, regularly spaced in time.

Wagenmakers, Farrell and Ratcliff [10] introduced a ‘shifting-strategy model’, which seemed able to generate 1/*f* fluctuations. This model assumes that the threshold level could evolve in time, as a consequence of the successive adoption of different strategies for controlling interval duration. Each strategy is characterized by a particular threshold that can be modeled by sampling uniformly from an interval centered on a baseline level. These successive strategies are employed during a limited and variable period of time (i.e., number of produced events). This shifting in strategy is modeled by sampling from a uniform distribution of usage times, bounded by a minimal (*d_min_*) and a maximal (*d_max_*) usage duration. Each iteration of the activation process is then realized until the reaching of a threshold T*_i_*: 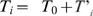
(2)where T_0_ represent the baseline threshold, and 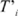 the deviation from this baseline, sampled from a uniform distribution of range *R*. These strategy shifts produce local plateaus in performance.

It is further assumed that the speed *v* with which activation grows over time is variable from one interval to the other, following an auto-regressive process of order one: 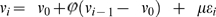
(3)where *v*_0_ represents the baseline speed, *ϕ* is the auto-regressive parameter, and *ε_i_* a centered white noise with unit variance. The time interval *C_i_* produced by iteration *i* is then simply given by
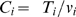
(4)


Delignières, Torre and Lemoine [11] showed that incorporating the shifting–strategy model in the *C* component of the Wing and Kristofferson model allowed to generate inter-tap interval series with similar short- and long-range correlation properties than experimentally observed. Note that the relative weight of the motor component in the Wing and Kristofferson model is controlled by multiplying motor delay terms by a constant λ.

**Figure 2:**
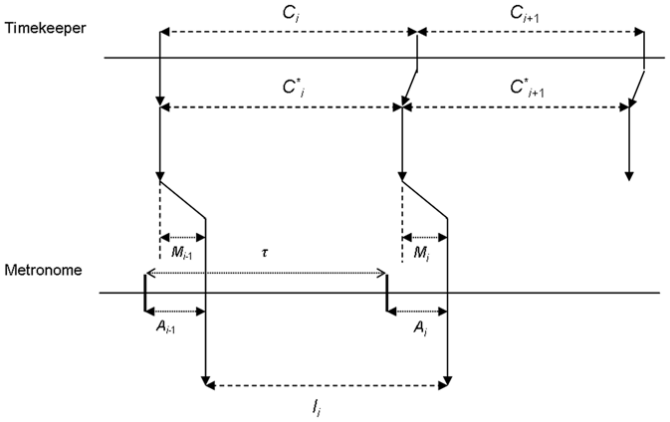
The Vorberg and Wing (1996)'s model for synchronization tapping. *C_i_*: interval produced by the timekeeper, C*_i_: interval adjusted by the linear phase correction process, *M_i_*: motor delay, *A_i_*: asynchrony to the metronome, *Ii*: effective interval produced by tapping, τ: fixed period of the metronome. We suppose in the present paper that *C_i_* presents fractal fluctuations.

The model proposed by Vorberg and Wing [3] for synchronization tapping is a little bit more complex. They started from a basic property of this kind of task, illustrated in Figure 2: each inter-tap interval (*I_i_*) corresponds to the difference between its previous and next asynchronies (*A_i_*_-1_ and *A_i_*), plus the period (*τ*) imposed by the metronome: 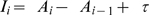
(5)


The main assumption of the model is that the preceding asynchrony is taken into account by a linear phase correction: The interval produced by the timekeeper is corrected by a fraction of the preceding asynchrony: 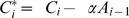
(6)


According to the Wing-Kristofferson model, the produced interval results from the combination of this corrected cognitive interval and the two successive motor delays: 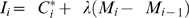
(7)


Combining Eq. (5), (6) and (7) leads to the following expression for current asynchrony: 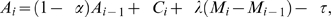
(8)


Torre and Delignières [7] showed that providing the timekeeper component (*C_i_*) of Eq. 8 using the shifting-strategy model allowed generating simulated series of asynchronies and inter-tap intervals presenting short- and long-range correlation similar to that experimentally observed.

**Figure 3:**
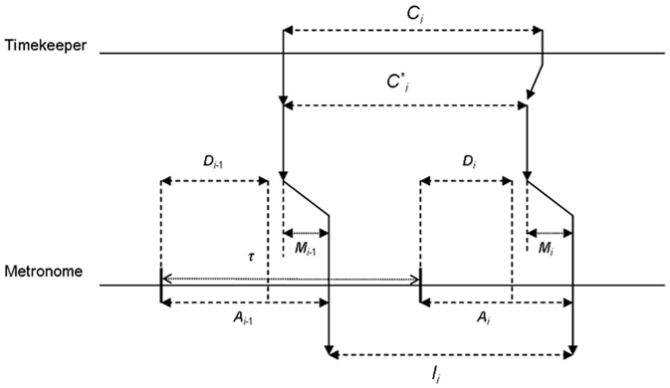
The model for syncopation tapping. *C_i_*: interval produced by the timekeeper, C*_i_: interval adjusted by the linear phase correction process, *M_i_*: motor delay, *D_i_*: estimated metronome half-period, *A_i_*: asynchrony to the metronome, *I_i_*: effective interval produced by tapping, τ: fixed period of the metronome. We suppose in the present paper that *C_i_* presents fractal fluctuations.



Let us try to extend Vorberg and Wing's model to account for syncopation. The main components of the proposed model are illustrated in Figure 3. We suppose that syncopation is based on a similar auto-regressive phase correction process as synchronization. Nevertheless, perceived asynchrony in this case is not, as previously, the time delay between the onset of the metronome and the effective tap, but, rather, the time between tap and an estimation of the duration of the half-period of the metronome. Let *D_i_* represent this estimation, for the tap completing the interval *I_i_*, and *A* ^*^ *_i_* the corresponding perceived asynchrony.

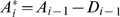
(9)


*A_i_* corresponding as previously to the ‘objective’ asynchrony between tap and metronome. The linear phase correction thus takes the following form: 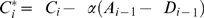
(10)


We assume that *D_i_* fluctuates around a baseline value (*τ*/2). Considering that *D_i_* represents the main innovation in this model, as compared with the previous synchronization model, we suppose that its successive fluctuations present a correlated structure. We argue that each estimation conserves the memory of the previous one, and as such we conceive the successive deviations (*d_i_*) from the baseline value as a random walk: 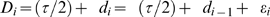
(11)where *ε_i_* is an uncorrelated white noise process. A simplest expression could be given by: 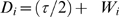
(12)where *W_i_* is ordinary Brownian motion. This modeling solution, however, suggests that *D_i_* is unbounded, which appears implausible. For correcting this problem we propose to bound *W_i_* within two limits (+*b* and −*b*). This bounding is likely to represent a kind of intermittent control of the estimation of the half-period, in order to maintain *D_i_* in an acceptable range. Combining Eq. (7), (10), and (12) leads to the following expression for asynchrony: 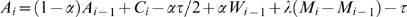
(13)


Additionally, we provide *C_i_* with fractal properties by means of the shifting-strategy model.

The aim of the present work was then to collect series of asynchronies and inter-tap interval in synchronization and in syncopation conditions, and to check whether the linear phase correction model (Eq. 8) and the present syncopation model (Eq. 13), both enriched by providing the timekeeper with fractal properties, were able to account for the correlation properties computed from empirical series. A basic requirement for this demonstration is obviously to use identical values for the set of parameters shared by the two models.

## Methods

Eleven participants (6 men and 5 women, mean age 30.8±7.1) took part in the experiment. None of them had extensive practice in music. They declared no particular competence involving specific coordination between the upper limbs, and no neurological injury or recent upper limb injury. They signed an informed consent form, and were not paid for their participation. The experimental protocol was approved by the Scientific Committee of the Faculty of Sport Science of University Montpellier I.

Participants were seated comfortably, their forearm, hand palm and other fingers resting on the table so that only the index finger of the dominant hand moved. Auditory signal were delivered at a constant frequency of 1.25 Hz. In the synchronization condition participants were instructed to keep the taps on the beep, and in the syncopation task to tap in between two adjacent beeps. In both case they were instructed to minimize the contact duration on the surface. Participants performed series of about 600 taps, corresponding to 8 minutes trials. Each participant performed two trials in each condition, and the order of the two conditions was counterbalanced within participants.

The auditory signals were generated by a PC-driven metronome. The taps were performed on a flat rectangular (4 cm×4 cm) pressure sensor fixed on a table and adjusted to the participants' comfort. The pressure data and metronome sequences were recorded with a sampling frequency of 300 Hz, using LabJack U12 device.

Analyses focused on asynchronies series. The times of the taps and the auditory signals were identified as the reaching of a threshold at each signal onset. Asynchronies were defined as the difference between the tap and the corresponding auditory signal.

### Simulation

The parameters of the shifting-strategy model were set as follows: T_0_ = 1600, *R = *20, *d_min_ = *1, *d_max_ = *100, *v_0_ = 2*, *ϕ* = 0.28, and *μ* = 0.09. The parameter controlling the weight of the motor component was set to *λ = *1.5. The additional parameters of the synchronization and syncopation models were set to *α* = 0.85 and *τ* = 800. Finally the bounding parameter for *W_i_* was *b* = 50. For both synchronization and syncopation, 100 series of 512 data points were simulated.

### 2.2 Data analysis

We first applied ARFIMA/ARMA modeling [10], [12] in order to evaluate the statistical evidence for the presence of genuine long-range correlations in experimental and simulated series. This method consists in fitting 18 models to the studied series: nine are ARMA (*p*,*q*) models, *p* and *q* varying systematically from 0 to 2, and the other nine are the corresponding ARFIMA (*p*,*d*,*q*) models, where *d* is the fractional integration parameter. The best model is selected using a goodness-of-fit statistic that is based on a trade-off between accuracy and parsimony. We used the Bayes Information Criterion (BIC) that was proven to give the best results in the detection of long-range dependence [12]. The ARFIMA/ARMA procedure provides two complementary criteria. The first one is the percentage of series that are better fitted by an ARFIMA model. The second is based on a transformation of the raw BIC values into weights (i.e. the probability that this model is the best over the set of candidate models; see [13]). We then computed the sum of the weights captured by the nine ARFIMA models, considering that the weights of all tested model sum to one.

In order to obtain an accurate assessment of long-range correlations in the series, we combined two methods: the Detrended Fluctuation Analysis in the time domain, and the Power Spectral Density method in the frequency domain. DFA [14] is based on the analysis of the relationship between the mean magnitude of fluctuations in the series and the length of the intervals over which these fluctuations are observed. The algorithm of DFA consists first in integrating the series *x*(*t*), and calculating for every *t* the cumulated sum of the deviations of the mean. This integrated series is then divided in non-overlapping intervals of length *n*. In each interval, a least squares line is fit to the data (representing the trend in the interval). The series is then locally detrended by subtracting to all values the theoretical value given by the regression. For each interval length *n*, the mean standard deviation [*F(n)*] of these integrated and detrended series is computed. For fractal series, a power law is expected, as *F(n)* ∝ *n* ^α^, α being the scaling exponent. α is estimated by the slope of the graph representing *F*(*n*) as a function of *n*, in log-log coordinates.

In the frequency domain we applied ^low^PSD_we_ [15], an improved version of the classical spectral analysis, including some preprocessing operations before the application of the Fast Fourier Transform (for details, see [15]). As proposed by the authors, we estimated the long-range behavior of series by computing the slope of the log-log power spectrum in the low-frequency region (*f*<1/8 of maximal frequency). We also assessed the sport-term behavior of series by computing the slopes in the high-frequency region (*f*>1/2 of maximal frequency).

We first analyzed the variability of experimental series by a two-way repeated measures ANOVA 2 (condition) X 2 (trial), in order to check whether results obtained in the two trials could be averaged in further analyses. DFA exponents and PSD slopes were then compared between conditions by means of one-way repeated measure ANOVAs. Finally experimental and simulated series were compared, for each variable and each condition, by one-way ANOVAs.

## Results

**Figure 4:**
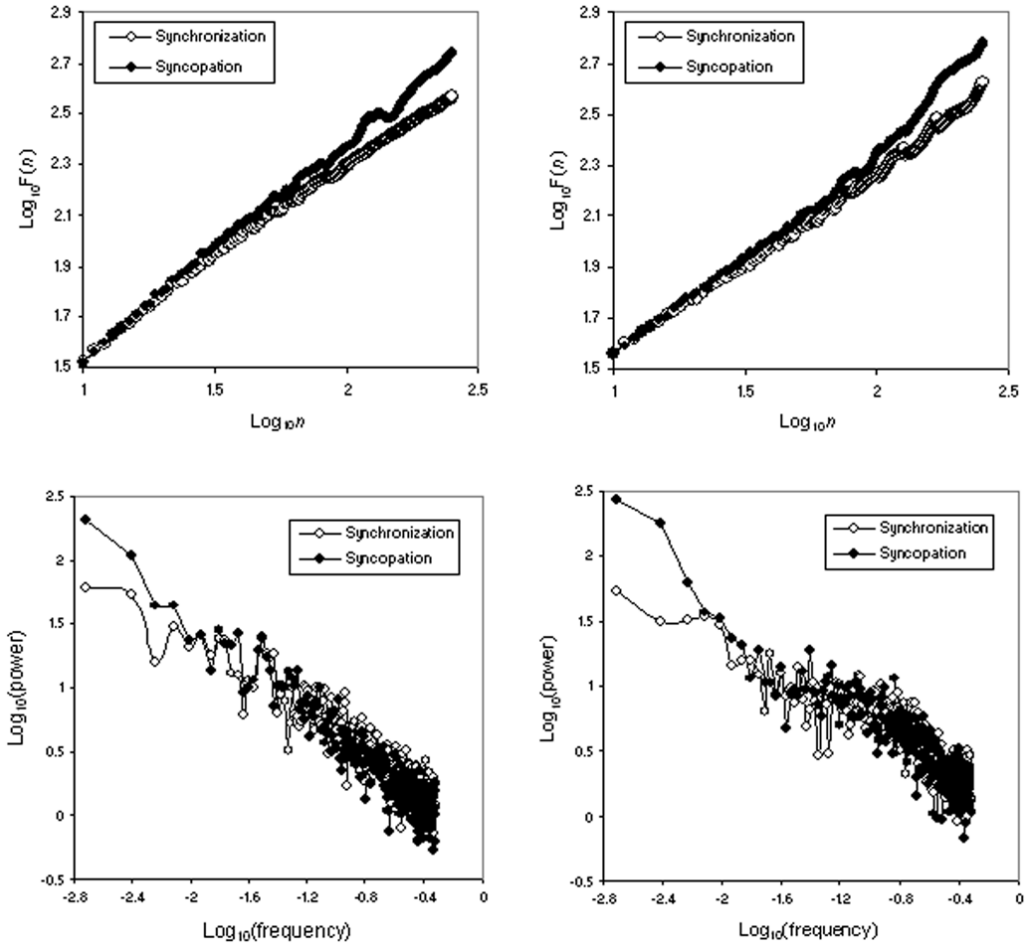
Times series analysis of asynchrony series. Upper panel: Detrended Fluctuation Analysis. Left: experimental series (averaged diffusion plot obtained by point-by-point averaging over the 10 participants; right: simulated series (averaged diffusion plots obtained by point-by-point averaging over 10 randomly selected simulated series). Lower panel: Power Spectral Density analysis: Left: experimental series (averaged log-log power spectra obtained by point-by-point averaging over the 10 participants; right: simulated series (averaged log-log power spectra obtained by point-by-point averaging over 10 randomly selected simulated series).

One participant was unable to adequately perform syncopation, and was excluded from analyses. Mean results, for experimental and simulated series, and for each condition, are presented in Table 1. The averaged diffusion plots (DFA) and power spectra (PSD) are reported in Figure 4.

**Table 1:** Numerical results of time series analyses for experimental and simulated series.

Series	Condition	Standard deviation	α DFA	PSD low frequency slope	PSD high frequency slope
Experimental (*N* = 10)	Synchronization	44.45 (±14.63)	0.73 (±0.10)	−0.60 (±0.69)	−0.90 (±0.64)
	Syncopation	48.25 (±19.56)	0.85 (±0.09)	−0.84 (±0.77)	−0.96 (±0.70)
Simulated (*N* = 100)	Synchronization	42.61 (±1.95)	0.75 (±0.08)	−0.73 (±0.57)	−0.91 (±0.52)
	Syncopation	49.47 (±3.88)	0.89 (±0.13)	−1.05 (±0.69)	−1.09 (±0.63)

Mean standard deviation of series, α exponent obtained from Detrended Fluctuation Analysis, low-frequency and high-frequency slopes obtained from Power Spectral Density analysis. Standard deviations in parentheses.

Experimental asynchronies presented a mean of −87 ms (±60) in synchronization, and 367 ms (±59) in syncopation. Variability was not significantly different between conditions (F(1,8) = 0.34, p = 0.576) and did not differ between the two successive trials (F(1,8) = 01.47, p = 0.260). The interaction was not significant (F(1,8) = 3.80, p = 0.087).

ARFIMA/ARMA modeling detected long-range correlation in 16 series over 18 in synchronization, and in 17 series over 18 in syncopation. The mean sum of ARFIMA weights was 0.89 in synchronization, and 0.95 in syncopation. These results attested for the effective presence of long-range dependence in asynchrony series, in both experimental conditions.

The mean α DFA exponent was significantly higher in syncopation than in synchronization (F(1,8) = 18.52, p = 0.003). This result confirms that reported by Chen et al. [8]. Accordingly, the mean low-frequency slope of PSD was significantly lower in syncopation than in synchronization (F(1,8) = 6.62, p = 0.033). There was no difference between conditions for the mean high-frequency slopes (F(1,8) = 0.58, p = 0.467).

Simulated asynchrony series presented a mean of 2 ms (±5) in synchronization, and 406 ms (±25) in syncopation. Note that mean asynchrony presented a lower variability in simulated than in experimental series. This was due to the fact that all simulated series were generated by the same set of parameters, in order to reproduce the mean features of experimental series. Considering series variability, simulated and experimental series presented similar mean standard deviations in the two conditions (synchronization: F(1,107) = 1.53, p = 0.220; syncopation = F(1,107) = 0.329, p = 0.567).

ARFIMA/ARMA modeling detected long-range correlations in 92 series over 100 in synchronization, and in 92 series over 100 in syncopation. The mean weight sum of ARFIMA weights was 0.89 in synchronization, and 0.95 in syncopation. These results, as previously, attested for the presence of genuine long-range dependence in simulated series.

Finally there was no difference in mean α DFA exponents between experimental and simulated series (synchronization: F(1,107) = 0.44, p = 0.507; syncopation = F(1,107) = 0.08, p = 0.776). There was as well no difference in mean low-frequency PSD slopes (synchronization: F(1,107) = 0.42, p = 0.521; syncopation = F(1,107) = 0.049, p = 0.824), nor in high-frequency slopes (synchronization: F(1,107) = 0.11, p = 0.743; syncopation = F(1,107) = 1.641, p = 0.203).

## Discussion

Our experimental results clearly replicated those of Chen et al. [8], with stronger correlations in syncopation than in synchronization asynchrony series. This result was attested by both DFA and PSD. We further showed by ARFIMA/ARMA modeling that in both cases series contained genuine long-range correlation.

The family of models presented in the introduction proposed a unifying framework for self-paced, synchronization and syncopation tapping. This modeling proposition is based on the now well-established distinction between event-based and emergent timing. This distinction has been supported by a number of studies, assessing performance variability [16], [17], [18], serial dependence [11], [19], neural correlates [20], [21], [22], or more theoretical perspectives [23], [24]. Event-based and emergent timing are supposed to be associated to the performance of discontinuous movements like tapping, and continuous movements like oscillations or circle drawing, respectively. While emergent timing is assumed to arise from the continuous regulation of non-temporal parameters (as oscillator stiffness) that determine movement frequency without needing any explicit representation of time, event-based timing is thought to involve an internal, explicit representation of temporal goals that is prescribed to the effectors independently of the motor execution itself. The models presented in this paper, involving a timekeeper entity, are representative of this event-based timing framework.

As expected, the synchronization model was able to generate asynchrony series reproducing the correlation properties of the corresponding experimental series, with a set of parameters similar to that used by Torre and Delignières [7]. ARFIMA modeling showed that these series contained long-range dependence. As pointed out by Torre and Delignières [7] and in accordance with the basic assumptions of the original model by Vorberg and Wing [3], this result strongly suggests the timekeeper that underlies the self-paced production of time intervals is still at work during synchronization to external signals.

We show in the present work that the addition of a simple process of estimation of the half-period of the metronome allows generating asynchrony series presenting the increase in serial correlations observed in syncopation experimental series. This rather psychologically plausible process adds a source of persistent correlation that combines with the previous one and seems sufficient to explain the observed difference in correlation strength. The examination of diffusion plots and power spectra shows that beyond the statistical equivalence of scaling exponents and slopes, experimental and simulated series share similar diffusion properties and frequency compositions.

We observed in the experimental series the typical negative mean asynchrony in synchronization (and correspondingly in syncopation a mean asynchrony inferior to the semi-period of the metronome). This anticipation tendency was already described in a number of experiments (for a review, see [25], [26]), and shows that taps are not performed in reaction to auditory signals, but rather as the result of internal prospective processes. The explanation of this anticipation tendency remains subject to debate [26], and our models are unable to reproduce this negative mean asynchrony [3], [7]. Chen et al. [8] proposed a model for synchronization tapping, composed of a hybrid oscillator coupled to a sine function modulated by a delayed version of actual movement that seemed able to generate this anticipation tendency. This model, nevertheless, remains focused on the synchronization condition, without any perspective of extension to self-paced or syncopation conditions. Moreover, it based on an oscillatory perspective, possessing a clear relevance in the case of emergent timing [11], but which has been clearly dismissed in the case of event-based timing, especially in tapping tasks [27]. Further efforts are needed, however, to account for the anticipation tendency in the present modeling framework.

The three present models share the presence of a timekeeping entity, considered a 1/*f* noise source. Note that the exact way we modeled this timekeeper (i.e. the shifting-strategy model) is not of central interest here. A number of mathematical solutions exist for generating this specific pattern of correlation that could have been used alternatively in the present context. The most important is that the same 1/*f* noise source, associated with motor delays, and if necessary of phase correction processes, could account for self-paced, synchronized or syncopated tapping performance [7].

The origin of fractal fluctuations in motor or cognitive performance is currently subject to debate, between the proponents of a so-called *nomothetic* approach, which conceive 1/*f* fluctuations as reflecting, at the macroscopic level, the complexity of the system that produced performance [28], and a *mechanistic* approach that suggests that 1/*f* fluctuations could arise from specific, local sources within the system [29]. The present models clearly support the second approach, with the presence of a fractal source (the timekeeper), that remains invariant across conditions and combines with others processes for producing the final sets of correlations in performance series.

Note that this timekeeping hypothesis should not be conceived as a claim for the existence of a single unit, within the central nervous system, in charge of time representation. The fractal fluctuations of the output of this timekeeper suggest rather that it represents a complex, distributed network within the CNS (Spencer, Zelaznik, Diedrichsen and Ivry [22] showed that the cerebellum plays an essential role in this network). In other words, our models claim for a *statistical* localization of the timekeeping process (i.e., its independence from implementation and phase correction processes), rather than for a *structural* localization in the brain.

## References

[1] Wing AM, Kristofferson AB (1973). The timing of interresponse intervals.. Percept Psychophys.

[2] Wing AM, Kristofferson AB (1973). Responses delays and the timing of discrete motor responses.. Percept Psychophys.

[3] Vorberg D, Wing AM, Keele S, Heuer H (1996). Modeling variability and dependence in timing.. Handbook of perception and action, vol 2.

[4] Gilden DL, Thornton T, Mallon MW (1995). 1/f noise in human cognition.. Science.

[5] Lemoine L, Torre K, Delignières D (2006). Testing for the presence of 1/*f* noise in continuation tapping data.. Can J Exp Psychol.

[6] Chen Y, Ding M, Kelso JAS (1997). Long memory processes (1/*f*
^α^ type) in human coordination.. Phys Rev Lett.

[7] Torre K, Delignières D (2008). Unraveling the finding of 1/f^β^ noise in self-paced and synchronized tapping: A unifying mechanistic model.. Biol Cyber.

[8] Chen Y, Ding M, Kelso JAS (2001). Origins of timing errors in human sensorimotor coordination.. J Mot Behav.

[9] Ivry RB (1996). The representation of temporal information in perception and motor control.. Curr Opin Neurobiol.

[10] Wagenmakers E-J, Farrell S, Ratcliff R (2004). Estimation and interpretation of 1/*f^α^* noise in human cognition.. Psychon Bull Rev.

[11] Delignières D, Torre K, Lemoine L (2008). Fractal models for event-based and dynamical timers.. Acta Psychol.

[12] Torre K, Delignières D, Lemoine L (2007). Detection of long-range dependence and estimation of fractal exponents through ARFIMA modeling.. Br J Math Stat Psychol.

[13] Wagenmakers E-J, Farrell S (2004). AIC model selection using Akaike weights.. Psychon Bull Rev.

[14] Peng CK, Havlin S, Stanley HE, Goldberger AL (1995). Quantification of scaling exponents and crossover phenomena in non stationary heartbeat time series.. Chaos.

[15] Eke A, Herman P, Bassingthwaighte JB, Raymond GM, Percival DB (2000). Physiological time series: distinguishing fractal noises from motions.. Pflügers Archiv.

[16] Robertson SD, Zelaznik HN, Lantero DA, Bojczyk G, Spencer RM (1999). Correlations for timing consistency among tapping and drawing tasks: evidence against a single timing process for motor control.. J Exp Psychol Hum Percept Perform.

[17] Zelaznik HN, Spencer RM, Doffin JG (2000). Temporal precision in tapping and circle drawing movements at preferred rates is not correlated: further evidence against timing as a general-purpose ability.. J Mot Behav.

[18] Zelaznik HN, Spencer RM, Ivry RB (2002). Dissociation of explicit and implicit timing in repetitive tapping and drawing movements.. J Exp Psychol Hum Percept Perform.

[19] Delignières D, Lemoine L, Torre K (2004). Time intervals production in tapping and oscillatory motion.. Hum Mov Sci.

[20] Ivry RB, Spencer RM, Zelaznik HN, Diedrichsen J (2002). The cerebellum and event timing.. Ann N Y Acad Sci.

[21] Spencer RMC, Ivry RB (2005). Comparison of patients with Parkinson's disease or cerebellar lesions in the production of periodic movements involving event-based or emergent timing.. Brain Cogn.

[22] Spencer RMC, Zelaznik HN, Diedrichsen J, Ivry RB (2003). Disrupted timing of discontinuous but not continuous movements by cerebellar lesions.. Science.

[23] Huys R, Jirsa VK, Studenka B, Rheaume N, Zelaznik HN, Fuchs A, Jirsa VK (2008). Human trajectory formation: taxonomy of movement based on phase flow topology.. Coordination: Neural, behavioral and social dynamics.

[24] Schöner G (2002). Timing, clocks, and dynamical systems.. Brain Cogn.

[25] Aschersleben G (2002). Temporal control of movements in sensorimotor systems.. Brain Cogn.

[26] Repp BH (2005). Sensorimotor synchronization: A review of the tapping literature.. Psychon Bull Rev.

[27] Torre K, Balasubramaniam R, Delignières D (2009). Oscillating in synchrony with a metronome: analysis of serial dependence and limit cycle dynamics.. Motor Control, in press.

[28] Kello CT, Beltz BC, Holden JG, Van Orden GC (2007). The emergent coordination of cognitive function.. J Exp Psychol Gen.

[29] Torre K, Wagenmakers EJ (2009). Theories and models for 1/*f* noise in human movement science.. Hum Mov Sci.

